# Shock: A presentation of enteric fever

**DOI:** 10.4103/0974-2700.66536

**Published:** 2010

**Authors:** Bilal Mirza, Lubna Ijaz, Muhammad Saleem, Muhammad Sharif, Afzal Sheikh

**Affiliations:** Department of Pediatric surgery, The Children’s Hospital and The Institute of Child Health, Lahore, Pakistan

Sir,

Enteric fever was known to have high rates of mortality and morbidity, even today, in many underdeveloped countries. The variability in its presentation, in pediatric as well as adult population makes the entity nonrecognizable at instances. This can result in maldiagnosis and mismanagement. We managed a case of enteric fever presented with shock due to massive bleeding per rectum. Bleeding per rectum in children is attributed, most of the times, to rectal polyp, rectal prolapse, anal fissure, and so on. Massive bleeding per rectum in children is rarely encountered.[1] Massive bleeding per rectum due to enteric fever–related ileal ulcers is not a common presentation of enteric fever in children. We present a case of 10-year-old boy presented to surgical emergency in shock.

A 10-year-old boy was received in the surgical emergency department of our institution in a condition of shock. Immediate brief review of the patient’s history pointed toward massive bleeding per rectum since 8 h as a cause of hypovolumic shock. There was a history of fever for 15 days, pain abdomen for 5 days, and vomiting for 2 days. At presentation, he was severely pale with cold extremities. Pulse was very weak with heart rate of 150/min, blood pressure was 60/nil, and respiratory rhythm was abnormal. He was not febrile at that time. Abdominal examination revealed tenderness in whole of the abdomen but more marked on the right side. Digital rectal examination revealed massive bleeding of mixed nature containing fresh as well as clotted and altered blood. The bleeding was so intense that within a few minutes, most of the part of the bed sheet got soaked with blood.

Immediately, 2 large bore I/V lines were maintained and crystalloid solution started infusing rapidly through one line. Luckily we got 2 pints of blood from the hospital blood bank. Blood was transfused and the patient got stabilized in 2–3 h. The patient was started on antibiotics, blood tests were requested, and octreotide infusion was started. In the mean time, the patient again got the same episode of massive bleeding per rectum and his vitals started deranging. Eight pints of blood had to be transfused to manage the massive blood loss. At one instance we had to perform cardiopulmonary resuscitation of the patient, but he recovered successfully from cardiac arrest. We were suspecting bleeding from Meckels’s diverticulum, then after arranging 3 pints of whole fresh blood, exploratory laparotomy was planned due to continuous massive bleeding per rectum and rapidly deteriorating condition of the patient.

At operation, the terminal 10 cm of ileum was inflamed. Mesenteric lymphadenopathy was also observed in that region. We performed limited right hemicolectomy, including the terminal 10 cm of ileum. On opening the affected part of the ileum, multiple bleeding ulcers were present on the mucosal surface, which was found to be the cause of massive bleeding per rectum. The resected specimen was sent for histopathology. Postoperative recovery was uneventful; and the patient was started orally on the 5^th^ postoperative day and discharged on the 7^th^ postoperative day on ciprofloxacin and paracetamol. His postoperative Widal test was strongly positive. Histopathology revealed enteric fever–related ulceration of the ileum extending up to the muscularis mucosae.

Enteric fever presents with a wide variety of systemic manifestations. There are as many as 16–30 million cases of enteric fever per year, almost exclusively in the developing world caused mainly by unhygienic conditions and poor sanitation, with a mortality rate of 10%. Typhoid fever may needs surgical interventions because of abdominal complications, such as intestinal perforation and bleeding, cholecystitis and gallbladder perforation, and pancreatitis; these represent the most serious complications of the illness. The ileum is mostly involved due to a high concentration and enlargement of the Payer’s patches leading to ulceration, bleeding, and perforation.[2]

The common causes of lower gastrointestinal tract (GIT) bleeding in children are polyps, Meckels’s diverticulum, trauma, inflammatory bowel disease, duplication cysts, vascular lesions, and infectious colitis.[1] Bleeding from enteric fever-related ulcers is very rare and few cases are reported in pediatric population.

The major cause of bleeding in enteric fever is deep intestinal ulceration. Intestinal hemorrhages usually occur after the second week. Local proliferation of salmonella organisms in the small intestinal lymph nodes and Payer’s patches ultimately leads to tissue necrosis and ulceration. When the process extends to erode a blood vessel, small intestinal hemorrhage ensues. The degree of bleeding may vary from microscopic bleeding in feces to massive bleeding, resulting in bad prognosis, if early surgical intervention has not been pursued.[3]

The diagnostic modalities to find out GIT bleeding are angiography, endoscopy, Meckels’s scan, and others.[3–5] These were not possible in the indexed case due to rapidly deteriorating condition of the patient. The diagnosis of enteric fever is concluded in our patient on the basis of history of fever, abdominal pain, and tenderness. This is further aided by Widal test, intraoperative findings, and histopathology.

The management options can be divided into conservative and interventional. Conservative management is for cases where bleeding is not massive and the patient is vitally stable. Conservative management includes gut rest by nothing per oral, intravenous fluids, fresh blood and fresh frozen plasma transfusion, antibiotics, proton pump inhibitors, and sometimes octreotide. In patients with massive bleeding that require multiple transfusions, early surgical intervention can save lives.[4–7]

Similarly, in our case the bleeding was massive and early surgical intervention proved boon for the patient. Resection of the part of intestine where bleeding ulcers were present proved curative.

We conclude that in settings where localized bleeding ulcers do not respond to conservative treatment, early surgical intervention can prove as a life-saving event for the patient.
